# Experiences of care for self-harm in the emergency department: the perspectives of patients, carers and practitioners

**DOI:** 10.1192/bjo.2022.35

**Published:** 2022-03-10

**Authors:** Jo Robinson, Eleanor Bailey

**Affiliations:** Orygen, Parkville, Victoria, Australia; and Centre for Youth Mental Health, University of Melbourne, Victoria, Australia; Orygen, Parkville, Victoria, Australia; and Centre for Youth Mental Health, University of Melbourne, Victoria, Australia

**Keywords:** Self-harm, suicide, emergency department, out-patient treatment, qualitative research

## Abstract

Emergency departments are often the point of entry to the healthcare system for people who self-harm, and these individuals are at high risk of further self-harm and suicide in the post-discharge period. These settings therefore provide a critical opportunity for intervention. However, many studies have identified that the experiences of patients, carers and the emergency department staff themselves is often suboptimal. In this editorial we summarise one such study, by O'Keeffe and colleagues, and consider strategies for improving the experiences of patients and their carers when presenting to the emergency department. We also reiterate the need for wider systemic change in attitudes and approaches towards people who self-harm that are pervasive across the healthcare system and beyond.

Emergency departments are often the first point of contact with the healthcare system for people engaging in self-harm, by which stage they may be experiencing a suicidal crisis. Indeed, the period immediately following discharge from emergency departments is associated with high risk of further self-harm and suicide.^1,2^ Emergency departments therefore present an important opportunity for intervention, not only because of the elevated levels of risk, but also because many of these individuals have sought help in the first place and may thus be more likely to engage with treatment. For these reasons, people who present to hospital following self-harm have been identified as a priority group when it comes to suicide prevention in many countries around the world.^3^

In their recent article published in *BJPsych Open*, O'Keeffe and colleagues report on a qualitative study examining experiences of care for self-harm in the emergency department from the perspective of patients, carers and practitioners.^4^ The study showed that, in general, the healthcare system is failing to meet the needs of people who present to the emergency department following self-harm. In the paper, the views of the different stakeholder groups are combined and four different themes are reported; here we summarise the findings as they relate to patients and carers, followed by those of practitioners.

## The perspectives of patients, carers and practitioners

From the perspective of patients, emergency departments were often viewed as the only way of accessing support, owing to the barriers they experienced in the community. However, the care provided in emergency departments tended to be experienced as invalidating and lacking in compassion and empathy. Patients and carers discussed feeling shame and guilt for being ‘time wasters’, sometimes as a direct result of the treatment they received. Risk assessments were perceived as a superficial ‘tick box exercise’ that failed to address ‘the root cause’ of distress and seemed to align with the requirements of the hospital rather than the needs of the patient. Carers described feeling excluded from decision-making, as well as ‘over-relied on’ yet ‘ill-equipped’ to keep the person in their care safe.

Practitioners agreed that the system is failing people who self-harm. This was largely attributed to: time constraints; pressure to prioritise the assessment, management and documentation of immediate risk; a lack of available services to refer to; and fear of being blamed if a patient subsequently died by suicide. Together these factors led to reduced capacity to develop rapport or express compassion and empathy. They also led to feelings of ‘powerlessness to help’, which in turn led to burnout and had a significant negative impact on their own mental health.

## The wider literature

Unfortunately, the challenge of delivering high-quality care to people who present to the emergency department following self-harm is not a new problem, and the study by O'Keeffe and colleagues adds to a growing body of literature that documents the challenges experienced by both patients and staff.^5–9^ Our own work with young people has shown that emergency departments are often found to be countertherapeutic, with staff appearing to lack both empathy and knowledge when treating young people who self-harm.^5^ This is significant as young people (and in particular young females) make up the majority of emergency department presentations for self-harm and rates of presentation in this population appear to have increased during the COVID-19 pandemic.^10,11^ This is also problematic given that negative experiences may reduce likelihood of future help-seeking. For example, Rosebrock and colleagues found that ~50% of patients attending an emergency department for a ‘suicidal crisis’ reported being unwilling to return, with negative experiences, less comprehensive assessments and longer waiting times all associated with unwillingness to return or attend a follow-up appointment.^6^

Despite highlighting predominantly negative experiences, research (including the current study) has identified numerous opportunities for improvement. These include the need for more personalised and compassionate care, more collaborative assessment practices, better staff training and increased access to aftercare services following discharge from the emergency department.^4,5,8^ These suggestions echo the recommendations made in current best practice guidelines (both in the UK, where the current study was conducted, and elsewhere), which cite the need for robust psychosocial assessment, appropriate mental health training for staff, and showing patients respect and compassion.^12,13^ However, despite the existence of such guidelines, many studies, including the one by O'Keeffe and colleagues, demonstrate that adherence to them is often suboptimal.^9,14,15^ In particular, research has focused on identifying and understanding poor adherence to the recommendation that every person who presents to the emergency department for self-harm should undergo a psychosocial assessment.^14,16,17^

## What is the solution?

### Changing emergency department culture and processes

There is clearly an urgent need for systemic and cultural change in emergency departments if we are to meet the needs of people who present following self-harm. According to O'Keeffe and colleagues this may be fostered through approaches targeting practitioners, patients and carers. Strategies targeting practitioners might include training to address stigma (among emergency department staff but also more broadly across the community), improved supervision practices and strategies to reduce burden on individual staff members in the unfortunate (yet at times unavoidable) event that a patient subsequently dies by suicide. Strategies targeting vicarious trauma in emergency department practitioners also have the capacity to reduce burnout and improve well-being, and might include structured debriefing or reflective practice with peers. For patients, O'Keeffe and colleagues highlight the need for evidence-based interventions and discuss the potential utility of adapting the Attempted Suicide Short Intervention Program (ASSIP), an intervention designed to foster hope through building the therapeutic alliance,^18^ to the emergency department setting. Collaborative safety planning, in which practitioners work with patients to create a written, personalised plan outlining warning signs and coping strategies, has also shown promise in emergency departments.^19^ More broadly (and in line with National Institute for Health and Care Excellence (NICE) guidelines and general good practice), applying the principles of compassionate care would also likely make a significant difference to the experiences of patients in the emergency department.^20^ Beyond approaches targeting patients and practitioners, it is crucial that supports are provided to carers. These individuals are frequently neglected, yet they are often tasked with the responsibility of keeping the patient safe; this may be particularly important for carers of younger patients.^21^

The physical environment of the emergency department is also often considered to be countertherapeutic to people presenting following self-harm or in a suicidal crisis;^5^ thus, there is scope to partner with patients and other stakeholders in order to improve some of the environmental characteristics of the emergency department (e.g. lighting), which in turn will likely lead to improved patient experiences.^22^ More radically, dedicated psychiatric emergency services (PES) (in other words, stand-alone emergency departments specifically for psychiatric patients) may serve to remove some of the challenges associated with care provided in the emergency department.^23^ PES units have been used with good effect in both the US and UK, and may also be worth pursuing elsewhere in the world. Finally, in addition to training for individual staff, strategies to improve adherence to best-practice guidelines in emergency departments should be adopted, including the active dissemination and promotion of guidelines by hospital leadership, developing protocols that clearly delineate roles and responsibilities, implementing decision support systems and ensuring that adequate time and resources are available to practitioners.^24^

### Access to services

Beyond emergency departments, there is a larger issue of service access. Indeed, it is acknowledged that there are risks associated with community-occurring self-harm (including risk of eventually presenting to the emergency department), yet there are barriers to accessing community-based mental health services. For example, long waiting lists, high levels of stigma and low visibility of available services often preclude people from accessing community-based support for self-harm in a timely manner. Aside from the need for extra funding for community-based services, a number of novel approaches have been developed to address this. For example some countries, including the UK, are developing alternatives to the emergency department; these are often referred to as ‘safe spaces’ and have a strong peer support component. Initial evaluations of these have shown that participants value the opportunity to develop supportive connections with peers and to discuss self-harm in an environment free of stigma.^25^ To date, however, limited evidence exists for their effectiveness. Another novel approach is the development of aftercare services. These are designed to deliver assertive follow-up care during the 3 months after presentation to the emergency department, with the aim of reducing suicide risk and increasing engagement with services. These are gaining traction in Australia, and early indications suggest that they are acceptable to patients and staff and that they appear to reduce rates of re-presentation to the emergency department.^26^ Robust evaluation is now required to assess their effectiveness, including their cost-effectiveness, and to ensure that they do not further fragment an already fragmented system of care. Of course, a patient's likelihood of engaging with aftercare services (or any other mental health service) is likely to be influenced by their experience in the emergency department, further emphasising the need to provide quality care at that first point of contact. Nonetheless, the broad implementation of new approaches such as safe spaces or aftercare services, combined with adequate funding for both primary and secondary healthcare services, would probably reduce pressure on emergency departments. This in turn would reduce the burden on practitioners and ultimately improve the experiences and treatment outcomes of patients.

### Stigma

Finally, it is acknowledged that stigmatising attitudes towards self-harm are widely held, in both the healthcare system and beyond. At a broader population level, approaches addressing this stigma, such as media campaigns or psychoeducation programmes delivered in educational settings, are required. Reduced stigma at a population level will likely have flow-on effects, including increased compassion on the part of health practitioners and improved well-being for people who self-harm.

## Conclusions

O'Keeffe and colleagues have added to a growing body of literature emphasising the urgent need for improved processes within emergency departments, as well as systemic changes to the wider healthcare system for the benefit of patients, carers and practitioners. Although this study does not necessarily reveal a new problem (indeed, research published over a decade ago highlights many of the same challenges^27^), it is timely. The COVID-19 pandemic has significantly and negatively affected people's mental health and, in turn, rates of self-harm and suicidal ideation; it has also placed unprecedented pressure on emergency departments and highlighted many of the pre-existing cracks in the healthcare system. Now more than ever we need to invest in the health of our community and particularly in those who may have previously been underserved by our healthcare system, including individuals presenting to emergency departments following self-harm, their families and carers, and the practitioners themselves.

## Data availability

Data availability is not applicable to this article as no new data were created or analysed in its preparation.

## References

[1] Hawton K, Bale L, Brand F, Townsend E, Ness J, Waters K, Mortality in children and adolescents following presentation to hospital after non-fatal self-harm in the multicentre study of self-harm: a prospective observational cohort study. Lancet Child Adolesc Health 2020; 4: 111–20. 31926769 10.1016/S2352-4642(19)30373-6

[2] Geulayov G, Casey D, Bale L, Brand F, Clements C, Farooq B, Suicide following presentation to hospital for non-fatal self-harm in the multicentre study of self-harm: a long-term follow-up study. Lancet Psychiatry 2019; 6: 1021–30. 31706930 10.1016/S2215-0366(19)30402-X

[3] World Health Organization. National Suicide Prevention Strategies: Progress, Examples and Indicators. WHO, 2018.

[4] O'Keeffe S, Suzuki M, Ryan M, Hunter J, McCabe R. Experiences of care for self-harm in the emergency department: comparison of the perspectives of patients, carers and practitioners. BJPsych Open 2021; 7(5): e175.

[5] Byrne SJ, Bellairs-Walsh I, Rice SM, Bendall S, Lamblin M, Boubis E, A qualitative account of young people's experiences seeking care from emergency departments for self-harm. Int J Environ Res Public Health 2021; 18(6): 2892. 33808995 10.3390/ijerph18062892 PMC8000083

[6] Rosebrock HY, Batterham PJ, Chen NA, McGillivray L, Rheinberger D, Torok MH, Nonwillingness to return to the emergency department and nonattendance of follow-up care arrangements following an initial suicide-related presentation. Crisis [Epub ahead of print] 22 Sep 2021. Available from: 10.1027/0227-5910/a000812. 34547919

[7] Koning KL, McNaught A, Tuffin K. Emergency department staff beliefs about self-harm: a thematic framework analysis. Community Ment Health J 2018; 54: 814–22. 29101707 10.1007/s10597-017-0178-8

[8] MacDonald S, Sampson C, Turley R, Biddle L, Ring N, Begley R, Patients’ experiences of emergency hospital care following self-harm: systematic review and thematic synthesis of qualitative research. Qual Health Res 2020; 30: 471–85. 31933427 10.1177/1049732319886566

[9] Fernandes J, Scheuermeyer FX, Chakraborty AT, Honer WG, Barbic D. What are Canadian emergency physicians’ attitudes and self-perceived competence toward patients who present with suicidal ideation? Can J Emerg Med 2021; 23: 668–72. 10.1007/s43678-021-00157-0 34196944

[10] Australian Institute of Health and Welfare. Suicide & Self-Harm Monitoring Data. Australian Government, 2021.

[11] Ougrin D, Wong B-C, Vaezinejad M, Plener PL, Mehdi T, Romaniuk L, Pandemic-related emergency psychiatric presentations for self-harm of children and adolescents in 10 countries (PREP-kids): a retrospective international cohort study. Eur Child Adolesc Psychiatry [Epub ahead of print] 7 Mar 2021. Available from: 10.1007/s00787-021-01741-6. PMC7937052 33677628

[12] Carter G, Page A, Large M, Hetrick S, Milner AJ, Bendit N, Royal Australian and New Zealand college of psychiatrists clinical practice guideline for the management of deliberate self-harm. Aust N Z J Psychiatry 2016; 50: 939–1000. 27650687 10.1177/0004867416661039

[13] National Institute for Health and Care Excellence. Self-Harm in Over 8s: Short-Term Management and Prevention of Recurrence. NICE, 2004. 31891461

[14] Pitman A, Tsiachristas A, Casey D, Geulayov G, Brand F, Bale E, Comparing short-term risk of repeat self-harm after psychosocial assessment of patients who self-harm by psychiatrists or psychiatric nurses in a general hospital: cohort study. J Affect Disord 2020; 272: 158–65. 32379609 10.1016/j.jad.2020.03.180

[15] Wilson MP, Kaur J, Blake L, Oliveto AH, Thompson RG, Pyne JM, Adherence to guideline creation recommendations for suicide prevention in the emergency department: a systematic review. Am J Emerg Med 2021; 50: 553–60. 34547697 10.1016/j.ajem.2021.07.042

[16] Quinlivan L, Gorman L, Littlewood DL, Monaghan E, Barlow SJ, Campbell S, ‘Wasn't offered one, too poorly to ask for one’ – Reasons why some patients do not receive a psychosocial assessment following self-harm: qualitative patient and carer survey. Aust N Z J Psychiatry [Epub ahead of print] 21 May 2021. Available from: 10.1177/00048674211011262. PMC8941717 34015945

[17] Cooper J, Steeg S, Bennewith O, Lowe M, Gunnell D, House A, Are hospital services for self-harm getting better? An observational study examining management, service provision and temporal trends in England. BMJ Open 2013; 3(11): e003444. 10.1136/bmjopen-2013-003444 PMC3840333 24253029

[18] Michel K, Gysin-Maillart A. ASSIP – Attempted Suicide Short Intervention Program: A Manual for Clinicians. Hogrefe Publishing, 2015.

[19] Stanley B, Brown GK, Brenner LA, Galfalvy HC, Currier GW, Knox KL, Comparison of the safety planning intervention with follow-up vs usual care of suicidal patients treated in the emergency department. JAMA Psychiatry 2018; 75: 894–900. 29998307 10.1001/jamapsychiatry.2018.1776 PMC6142908

[20] Cole-King A, Green G, Gask L, Hines K, Platt S. Suicide mitigation: a compassionate approach to suicide prevention. Adv Psychiatr Treat 2013; 19: 276–83.

[21] Byrne S, Morgan S, Fitzpatrick C, Boylan C, Crowley S, Gahan H, Deliberate self-harm in children and adolescents: a qualitative study exploring the needs of parents and carers. Clin Child Psychol Psychiatry 2008; 13: 493–504. 18927136 10.1177/1359104508096765

[22] Liddicoat S. Designing a supportive emergency department environment for people with self harm and suicidal ideation: a scoping review. Aust Emerg Care 2019; 22: 139–48. 10.1016/j.auec.2019.04.006 31129061

[23] Zeller SL. Treatment of psychiatric patients in emergency settings. Prim Psychiatry 2010; 17: 35–41.

[24] Fischer F, Lange K, Klose K, Greiner W, Kraemer A. Barriers and strategies in guideline implementation – a scoping review. Healthcare 2016; 4(3): 36. 27417624 10.3390/healthcare4030036 PMC5041037

[25] Boyce M, Munn-Giddings C, Secker J. ‘It is a safe space’: self-harm self-help groups. Ment Health Rev 2018; 23: 54–63.

[26] Wright AM, Lee SJ, Rylatt D, Henderson K, Cronje H-M, Kehoe M, Coordinated assertive aftercare: measuring the experience and impact of a hybrid clinical/non-clinical post-suicidal assertive outreach team. J Affect Disord Rep 2021; 4: 100133.

[27] Taylor TL, Hawton K, Fortune S, Kapur N. Attitudes towards clinical services among people who self-harm: systematic review. Br J Psychiatry 2009; 194: 104–10. 19182168 10.1192/bjp.bp.107.046425

